# Reduction of 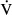
_O2_ slow component by priming exercise: novel mechanistic insights from time-resolved near-infrared spectroscopy

**DOI:** 10.14814/phy2.12432

**Published:** 2015-06-24

**Authors:** Yoshiyuki Fukuoka, David C Poole, Thomas J Barstow, Narihiko Kondo, Masato Nishiwaki, Dai Okushima, Shunsaku Koga

**Affiliations:** 1 Environmental Physiology Laboratory, Prefectural University of KumamotoKumamoto, Japan; 2 Graduate School of Health and Sports Science, Doshisha UniversityKyoto, Japan; 3 Departments of Anatomy and Physiology and Kinesiology, Kansas State UniversityManhattan, Kansas; 4 Graduate School of Cultural Studies and Human Science, Kobe UniversityKobe, Japan; 5 Faculty of Engineering, Osaka Institute of TechnologyOsaka, Japan; 6 Applied Physiology Laboratory, Kobe Design UniversityKobe, Japan

**Keywords:** Heavy exercise, hemoglobin concentration, muscle microcirculation, muscle O_2_ diffusing capacity

## Abstract

Novel time-resolved near-infrared spectroscopy (TR-NIRS), with adipose tissue thickness correction, was used to test the hypotheses that heavy priming exercise reduces the V̇_O2_ slow component (V̇_O2SC_) (1) by elevating microvascular [Hb] volume at multiple sites within the quadriceps femoris (2) rather than reducing the heterogeneity of muscle deoxygenation kinetics. Twelve subjects completed two 6-min bouts of heavy work rate exercise, separated by 6 min of unloaded cycling. Priming exercise induced faster overall V̇_O2_ kinetics consequent to a substantial reduction in the V̇_O2SC_ (0.27 ± 0.12 vs. 0.11 ± 0.09 L·min^−1^, *P* < 0.05) with an unchanged primary V̇_O2_ time constant. An increased baseline for the primed bout [total (Hb + Mb)] (197.5 ± 21.6 vs. 210.7 ± 22.5 *μ*mol L^−1^, *P* < 0.01), reflecting increased microvascular [Hb] volume, correlated significantly with the V̇_O2SC_ reduction. At multiple sites within the quadriceps femoris, priming exercise reduced the baseline and slowed the increase in [deoxy (Hb + Mb)]. Changes in the intersite coefficient of variation in the time delay and time constant of [deoxy (Hb + Mb)] during the second bout were not correlated with the V̇_O2SC_ reduction. These results support a mechanistic link between priming exercise-induced increase in muscle [Hb] volume and the reduced V̇_O2SC_ that serves to speed overall V̇_O2_ kinetics. However, reduction in the heterogeneity of muscle deoxygenation kinetics does not appear to be an obligatory feature of the priming response.

## Introduction

Exercise performed above the lactate threshold in the heavy domain evinces a V̇_O2_ slow component (V̇_O2SC_), superimposed upon the primary (fast) V̇_O2_ kinetics, that elevates pulmonary and muscle V̇_O2_. This “extra” V̇_O2_, which may exceed 1000 mL·min^−1^, slows overall V̇_O2_ kinetics (Gerbino et al. 1996) and is associated with compromised exercise tolerance (Murgatroyd and Wylde 2011; Grassi et al. 2015). Heavy priming exercise substantially reduces the V̇_O2SC_ contribution to the achieved V̇_O2_ in the presence or absence of speeded fast component kinetics (Bangsbo et al. 2000; Rossiter et al. 2001; Tordi et al. 2003; Paterson et al. 2005; Sahlin et al. 2005; Poole and Jones 2012).

Resolution of the mechanistic bases for priming exercise-induced V̇_O2SC_ reduction will significantly improve our understanding of muscle energetics. In this regard, current theories for the etiology of the reduced V̇_O2SC_ include increased bulk and local blood flow and O_2_ delivery (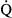
_O2_) via residual vasodilation and acidemia-induced rightward shift of the hemoglobin (Hb) O_2_ dissociation curve (Poole et al. 1991; Saitoh et al. 2009; Jones et al. 2011). These effects would (presumably) serve to improve matching of 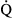
_O2_-to-V̇_O2_ thereby raising muscle and microvascular oxygen pressure (PO_2_) and enhancing blood-myocyte O_2_ flux and mitochondrial control by raising intracellular PO_2_ (McDonough et al. 2005). In support of this contention, several previous studies carried out by conventional (continuous wave, CW) near-infrared spectroscopy (NIRS) observed a priming exercise-induced slowing of muscle deoxygenation (i.e., [deoxy (Hb + Mb)]) kinetics (Rossiter 2011) and a reduced [deoxy (Hb + Mb)] at the baseline of the second exercise bout (Spencer et al. 2012). However, multichannel CW-NIRS found that the reduction in spatial heterogeneity across 10 sites in the quadriceps femoris muscle(s) did not correlate with the priming exercise-induced decrease in the V̇_O2SC_ (Saitoh et al. 2009), suggesting that improved matching of 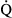
_O2_-to-V̇_O2_ was not responsible. One striking limitation of CW-NIRS is that it assesses relative rather than absolute [deoxy (Hb + Mb)] and thus exercise-induced alterations and variability in the optical physics (i.e., absorbance, scattering, path length) and also adipose tissue thickness (ATT) among subjects may have obscured the underlying response(s) (Koga et al. 2011). Consequently, it remains unknown to what extent prior heavy exercise influences the temporal and spatial profiles of the absolute [deoxy (Hb + Mb)] (ATT corrected) (Bowen et al. 2013) and consequently the mean muscle PO_2_ (Koga et al. 2012) of the different muscle regions during subsequent heavy exercise.

To circumvent these problems we have established a state-of-the art method to quantify absolute [deoxy (Hb + Mb)] and its spatial heterogeneity within the quadriceps femoris (QF) muscle during cycle exercise using time-resolved (TR) NIRS with ATT correction (Chin et al. 2011; Koga et al. 2011, 2014; Bowen et al. 2013). Moreover, based upon our current understanding of muscle O_2_ diffusion where the muscle O_2_ diffusing capacity (D_m_O_2_) is determined by the number/volume of red blood cells (RBCs) in the capillary bed adjacent to muscle fibers at any instant (Federspiel and Popel 1986; Groebe and Thews 1990), we hypothesized that priming exercise-induced increases in [total (Hb + Mb)] (Burnley et al. 2002; DeLorey et al. 2007; Jones et al. 2008) would relate quantitatively to V̇_O2SC_ reductions. In addition, if this represents a crucial mechanism for improving myocyte O_2_ delivery and metabolic control, decreased 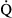
_O2_-to-V̇_O2_ heterogeneity would not be an obligatory facet of the reduced V̇_O2SC_ response. Accordingly, using TR-NIRS across multiple QF muscle sites during primed heavy exercise we tested the following hypotheses: (1) The V̇_O2SC_ would be reduced in proportion to the greater [total Hb] volume present. (2) The faster overall V̇_O2_ kinetics would not be dependent upon a reduction in the heterogeneity of the absolute [deoxy (Hb + Mb)] kinetics.

## Methods

### Subjects

Twelve physically active healthy male subjects (age, 23.4 ± 4.3 years; height, 173.8 ± 4.8 cm; and weight, 62.5 ± 6.3 kg) participated in this study. The study was approved by the Human Subjects Committee of Kobe Design University, in accordance with the Declaration of Helsinki. Explanation of the experimental protocol, including any possible risks and benefits associated with the testing procedures, were discussed with the subjects, and written consent was obtained from all subjects before voluntary participation in this study.

### Correction of amplitude of muscle deoxygenation for adipose tissue thickness

Measurement of ATT under each optode site over the vastus lateralis (VL) and the rectus femoris (RF) muscles at the distal- and proximal sites was made by B-mode ultrasound (model Logiq 400; GE-Yokogawa Medical Systems, Japan) with the subject in an upright position (Chin et al. 2011; Koga et al. 2011). In order to quantify the influence of ATT on the NIRS signal (Niwayama et al. 2000; Koga et al. 2011), we applied a linear regression of the relationship between [total (Hb + Mb)] and ATT that was established by a previous report (Bowen et al. 2013). Resting [total (Hb + Mb)] was determined at each site from a 2 min resting average in the upright-seated position. Measured [deoxy (Hb + Mb)] and [total (Hb + Mb)] values at each individual muscle site were corrected to a common ATT of 0 mm on this regression of [total (Hb + Mb)] and ATT. This normalization process allowed absolute values of both [deoxy (Hb + Mb)] and [total (Hb + Mb)] to be compared between subjects and muscle sites differing in ATT.

### Exercise tests

On each data-collection day, subjects reported to the laboratory at least 2 h after their last meal. They were asked to avoid caffeine and alcohol ingestion and strenuous exercise for 24 h before the test. The temperature and relative humidity of the laboratory were maintained at 25°C and 50%, respectively. On the first visit, seat height and handlebar position on an electronically braked cycle ergometer (Combi 232C, Tokyo, Japan) were recorded and reproduced on subsequent tests.

### Incremental exercise tests

The first visit was used to familiarize the subjects with testing procedures and to determine the peak V̇_O2_, gas exchange threshold (GET), and work rates for the constant work-rate tests. The protocol, which was designed to produce volitional exhaustion within 10–15 min, consisted of 4 min of unloaded exercise, followed by work rate increases of 25–30 W·min^−1^ until the limit of tolerance (Chin et al. 2011; Koga et al. 2011). Pedaling frequency was held constant at 60 rpm for all exercise bouts with the aid of an audible metronome. The peak V̇_O2_ was defined as the highest V̇_O2_ achieved during the test averaged over a 20-sec interval. The GET was estimated from gas exchange measurements using the V-slope method, ventilatory equivalents, and end-tidal gas tensions (Beaver et al. 1986).

### Constant work-rate exercise tests

Square-wave exercise transition tests were conducted on separate days. Each constant work-rate exercise test was performed for 6 min. The work rates for heavy exercise were calculated to elicit 40% of the difference between the GET V̇_O2_ and peak V̇_O2_ [Δ40 V̇_O2_ = GET V̇_O2_ + 0.4 (peak V̇_O2_ − GET V̇_O2_)], based on the V̇o_2_/work rate with account taken of the lag in V̇o_2_ relative to the work rate during ramp exercise. Over the next two visits, repeated bouts of heavy cycling exercise were performed twice at approximately the same time of day, with 3 days between visits. Moreover, a subset of four subjects performed, on separate days, an additional heavy exercise bout designed to elicit 20% of the difference between the GET V̇_O2_ and peak V̇_O2_ (Δ20 V̇_O2_). The constant work-rate exercise protocol consisted of 1 min of rest and 4 min of unloaded exercise, followed by two 6-min bouts of heavy exercise separated by 6 min of unloaded exercise at a pedal frequency of 60 rpm.

### Measurements

#### Pulmonary V̇_O2_

Subjects breathed through a low-resistance hot-wire flowmeter for measurement of inspiratory and expiratory flows. The flowmeter was calibrated repeatedly by inputting known volumes of room air at various mean flows and flow profiles. Expired oxygen and carbon dioxide concentrations were determined by gas analysis (Minato-Medical AE-300S, Japan) from a sample drawn continuously from the mouthpiece. Alveolar gas exchange variables were calculated breath-by-breath gas exchange measurement system (Minato-Medical AE-300S, Japan) (Beaver et al. 1981) from time aligned gas volume and concentration signals.

#### Muscle deoxygenation

The absolute muscle [deoxy (Hb + Mb)], [oxy (Hb + Mb)], and [total (Hb + Mb)] profiles at 4 sites in the quadriceps of the dominant leg were measured by two time-resolved spectroscopy (TRS) NIRS systems (TRS-20 each with two channels, Hamamatsu Photonics K.K., Japan). This system measures the distribution of in vivo optical path lengths, thereby enabling the determination of absolute [Hb + Mb] concentration (*μ*mol L^−1^). Previous studies showed that the deoxygenation measured by the TRS correlated significantly with the oxyhemoglobin saturation in both the blood and a purified-hemoglobin phantom solution (Hamaoka et al. 2000).

The optodes were housed in black rubber holders that helped to minimize extraneous movement, thus ensuring that the position of the optodes was fixed and invariant. The distal optodes were placed on the lower third of the VL and the RF muscles parallel to the major axis of the thigh. The location of the distal optodes on the VL muscle was chosen to represent the single site NIRS measurement conducted by previous studies (e.g., (Ferreira et al. 2007, 2005; Wilkerson et al. 2004)). The proximal optode pairs on the VL and the RF muscles were located ∽10–15 cm from the distal optode pairs. The interoptode spacing between emitter and receiver was 3 cm. The depth of the measured area was assumed to be approximately half of the distance between the emitter and the receiver, ∽1.5 cm. The skin under the probes was carefully shaved. Pen marks were made on the skin to indicate the margins of the rubber holder to check for any downward sliding of the probe during cycling and for accurate probe repositioning on subsequent days. No sliding was observed in any subject at the end of each protocol. The principles of operation and algorithms utilized by the equipment have been described in detail elsewhere (Oda et al. 1999; Ohmae et al. 2006). Briefly, the TRS system consists of a pico-second (ps) light pulser that emits three wavelengths (760, 795, and 830 nm), with a repetition frequency of 5 MHz and a full width at half maximum (FWHM) of 100 ps. A time-correlated single-photon counting board was installed to acquire temporal profiles of photons. The laser diodes and photomultiplier tube are connected to a lightweight plastic probe by optical fibers for single-photon detection. The time-correlated single-photon counting board is used for the parallel acquisition of time-resolved reflectance curves. In the present study, estimation of the optical path length, reduced scattering coefficient (*μ*_s_’) and absorption coefficient (*μ*_a_) are achieved by fitting the receiver profile of photon counts over time to a function based on diffusion theory (Oda et al. 1999; Ohmae et al. 2006). The output frequency was selected as 0.5 Hz. We reasoned that, as [Mb] would not be expected to change within the sampled volumes, any increased [Hb + Mb] reflected that of [Hb] (Davis and Barstow 2013). Validation of the equipment was performed before each test, by measuring the instrument's responses when the input and receiving fibers faced each other through a neutral-density filter in a black tube.

### Data analysis

Individual responses of pulmonary V̇_O2_ and [deoxy (Hb + Mb)] during the baseline (BL)-to-exercise transitions were time-interpolated to 1-sec intervals, and averaged across each transition for each subject. The response curve of V̇_O2_ was fit by a three-term exponential function (Eq. 2) that included amplitudes, time constants, and time delays, using nonlinear least-squares regression techniques (Gerbino et al. 1996; Fukuba et al. 2002; Grassi et al. 2003; Ferreira et al. 2005). The computation of best-fit parameters was chosen by the program (Kaleida Graph) so as to minimize the sum of the squared differences between the fitted function and the observed response.

The first exponential term started with the onset of exercise and the second and third terms began after independent time delays (Ma et al. 2010).



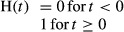




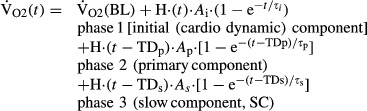
1

where the subscripts *i*, *p*, and *s* refer to initial, primary, and slow components, respectively; V̇_O2_ (BL) is the unloaded exercise baseline value; *A*_i_, *A*_p_, and *A*_s_ are the asymptotic amplitudes for the exponential terms; *τ*_i_, *τ*_p_, and *τ*_s_ are the time constants; and TD_P_, and TDs are the time delays. Mean response time (MRT_p_) for the primary phase of V̇_O2_ was defined as the sum of TD_p _+ *τ*_p_. The phase I V̇_O2_ at the start of phase II (i.e., at TD_P_) was assigned the value for that time (*A*_i_′). The physiologically relevant amplitude of the primary exponential component during phase II (*A*_p_′) was defined as the sum of *A*_i_′ + *A*_p_. Because of concerns regarding the validity of using the extrapolated asymptotic value for the SC (*A*_s_) for comparisons, we used the value of the slow exponential function at the end of exercise, defined as *A*_s_′. Alternatively, the slow component of V̇_O2_ was calculated as the change from 3 to 6 min (V̇_O2_(6-3)).

Subsequently, absolute [deoxy (Hb + Mb)] data were then fit from the time of initial and primary increases in [deoxy (Hb + Mb)] to 120 s with a two-exponential model of the form in Eq. 3 to determine the time course of muscle deoxygenation.



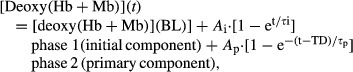
2

where the subscripts i and p refer to initial and primary components, respectively; [deoxy (Hb + Mb)] (BL) is the unloaded exercise baseline value; *A*_i_ and *A*_p_ are the symptotic amplitudes for the exponential terms; *τ*_i_ and *τ*_p_ are the time constants; and TD is the initial component duration from the onset of exercise to the onset of primary component of [deoxy (Hb + Mb)]. The absolute primary amplitude (*A*_a_) was defined as the sum of *A*_p_ + BL. The TD and *τ*_p_ of the [deoxy (Hb + Mb)] response were summed (MRT_p_) to provide an indication of the overall dynamics of the primary component (Koga et al. 2007).

The averaged slow components of both the [deoxy (Hb + Mb)] and [total (Hb + Mb)] (i.e., [deoxy (Hb + Mb)](6-3) and [total (Hb + Mb)](6-3)) were calculated as the changes from 3 to 6 min across the four sites. The priming-induced reductions in both [deoxy (Hb + Mb)](6-3) and [total (Hb + Mb)](6-3) from the first bout to the second bout were calculated to analyze the correlation with the reduced slow component of V̇_O2_ (V̇_O2_(6-3)).

### Statistics

Data are presented as mean ± SD. Intersite coefficient of variation [CV (%); 100·SD/mean of the four sites values] for each subject was calculated to show spatial heterogeneity of the amplitude and kinetic profiles of the muscle deoxygenation (Saitoh et al. 2009). When the comparison for differences between the first and the second bouts was justified, a paired t-test was used. Significance was accepted when *P* < 0.05. A two-way ANOVA [sites (distal/proximal) and muscles (VL/RF)] was performed to evaluate significant differences in the muscle deoxygenation profiles. When a significant difference was detected, this was further examined by post hoc *Scheffe* test. The relationship between two variables was analyzed by the correlation or linear regression analysis.

## Results

Peak V̇_O2_/body mass reflected the active lifestyle for these subjects (52.5 ± 6.2 mL·min^−1^·kg^−1^). The average work rates at the GET + Δ40 and Δ20 were 183 ± 25 W and 163 ± 20 W, respectively.

### Pulmonary O_2_ uptake kinetics

**Figure 1:**
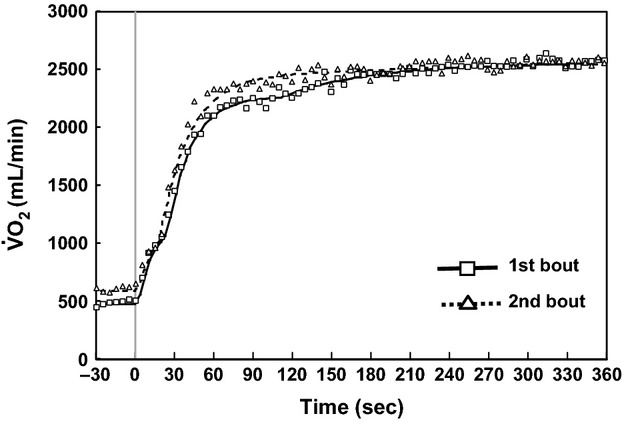
Mean pulmonary O_2_ uptake responses to the first (square) and second (triangle) bouts of heavy cycling exercise along with the best fitting three-term exponential function.

Priming increased baseline V̇_O2_ and substantially reduced the V̇_O2SC_ (*A*_s_′) 59% (both *P* < 0.05, Fig.1 and Table1) without altering end-exercise V̇_O2_. In addition, priming caused a significant shortening of the primary component time delay (TD_p_, *P* < 0.05) and consequently the mean response time (MRT_p_, ∽7%, *P* < 0.05) but did not change either the time constant (*τ*_p_) or the amplitude (*A*_p_′, phase I + phase II).

**Table 1:** Parameters of pulmonary V̇_O2_ kinetics following the onset of two sequential bouts of heavy exercise.

	1st bout	2nd bout
BL, L·min^−1^	0.47 ± 0.09	0.58 ± 0.08*
TD_p_, *s*	20.9 ± 7.4	16.8 ± 4.2*
*τ*_p_, *s*	21.1 ± 6.5	22.5 ± 7.4
MRT_p_, *s*	42.1 ± 6.2	39.3 ± 7.1*
*A*_p_’, L·min^−1^	1.88 ± 0.33	1.79 ± 0.33
*A*_a_, L·min^−1^	2.35 ± 0.31	2.37 ± 0.31
TD_s_, *s*	143.4 ± 64.7	176.1 ± 86.8
*A*_s_’, L·min^−1^	0.27 ± 0.12	0.11 ± 0.09*
V̇_O2_(6-3), L·min^−1^	0.22 ± 0.13	0.10 ± 0.03*

Values are mean ± SD.

V̇_O2_, O_2_ uptake

BL, baseline

TD_p_, time delay of phase II

*τ*_p_, time constant of phase II

MRT_p_, mean response time of the primary component of the response (MRT_p_ = TD_p_ + *τ*_p_)

*A*_p_’, amplitude of phase I + phase II, not including BL

*A*_a_, absolute primary amplitude (BL + *A*_p_’)

TD_s_, time delay of phase III (slow component)

*A*_s_’, amplitude of phase III

V̇_O2_(6-3), the change in V̇_O2_ from 3 min to 6 min.

* *P* < 0.05, compared to 1st bout.

### [total (Hb + Mb)]

**Figure 2:**
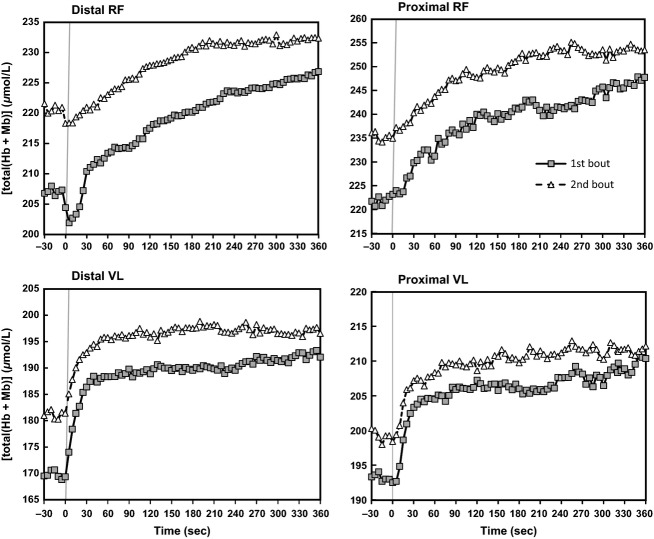
Adipose tissue thickness corrected absolute mean concentrations of total hemoglobin/myoglobin [total (Hb + Mb)] response at each site between first bout (square) and second bout (triangle) of heavy exercise using mean values of all subjects (please note that error bars are omitted for clarity). The baseline of [total (Hb + Mb)] response at each site before the second bout of exercise was significantly higher than for the first bout of exercise (*P* < 0.05).

As shown in the [total (Hb + Mb)] profiles at each site in Figure2, the averaged baseline [total (Hb + Mb)] response across the four muscle sites after priming was significantly greater than for the first bout of exercise (197.5 ± 21.6 vs. 210.7 ± 22.5 *μ*mol L^−1^, *P* < 0.01). The averaged end-exercise [total (Hb + Mb)] across the four muscle sites after priming was also significantly elevated (218.8 ± 22.8 vs. 225.7 ± 24.0 *μ*mol L^−1^, *P* < 0.05), while the averaged amplitude of [total (Hb + Mb)] was significantly reduced after priming (23.3 ± 3.0 vs. 15.1 ± 2.0 *μ*mol L^−1^, *P* < 0.05). Specifically, at both proximal and distal RF sites the end-exercise values of the [total (Hb + Mb)] response were increased after priming, (*P* < 0.05) whereas those for the VL muscle were not (proximal, *P* = 0.083; distal, *P* = 0.081) despite a significantly greater baseline (*P* < 0.05).

**Figure 3:**
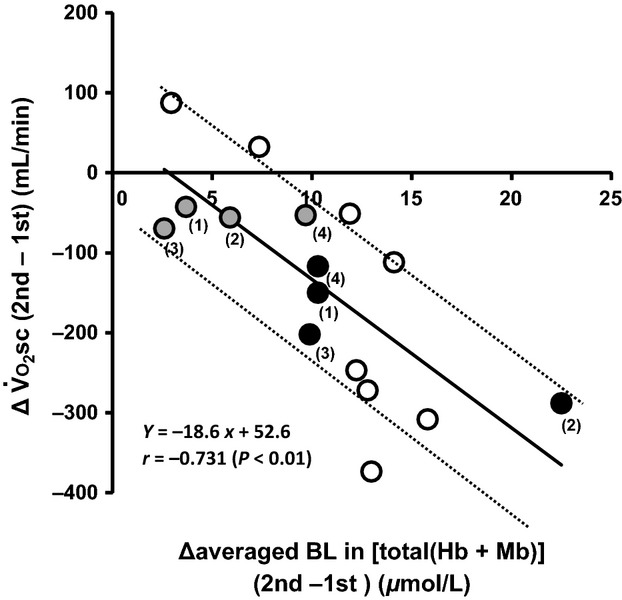
Relationship between the priming-induced reduction in V̇_O2_ slow component and the increased [total (Hb + Mb)] at baseline across the four sites (*r* = −0.731, *P* < 0.01). Dotted lines indicate the 95% confidence interval. Open circles indicate subjects who only performed at Δ40V̇_O2_ exercise intensity whereas gray and black circles are the four subjects who performed heavy exercise at both Δ20V̇_O2_ and Δ40V̇_O2_. The number from 1 to 4 is each subject's number.

At the Δ40V̇_O2_ (GET + Δ40) exercise intensity the reduced V̇_O2SC_ (Table1) was correlated with an increased BL of [total (Hb + Mb)] across the four muscle sites (*r* = −0.720, *P* < 0.01). As expected, for Δ20V̇_O2_ (GET + Δ20, *n* = 4) there was a much smaller reduction of V̇_O2SC_ compared with Δ40V̇_O2_ (i.e., ∽60 vs. 160 mL·min^−1^, *P* < 0.05). For both Δ20 V̇_O2_ and Δ40 V̇_O2_ data sets the reduced V̇_O2SC_ was correlated significantly with the increased [total (Hb + Mb)] BL across the four muscle sites (*r* = −0.731, *P* < 0.01, Figure3, 95% confidential interval, ±50.4 mL·min^−1^). In contrast, the reduction in [total (Hb + Mb)](6-3) or reduced amplitude of [total (Hb + Mb)] response from the first bout to the second bout was not related to the V̇_O2_(6-3) reduction from the first bout to the second bout (*r* = −0.228, *P* = 0.472; *r* = 0.06, *P* = 0.853, respectively). Only the increased BL in [total (Hb + Mb)] was clearly shown after priming to be related to the reduction of V̇_O2SC_.

### [Deoxy (Hb + Mb)] kinetics and their spatial heterogeneity

**Figure 4:**
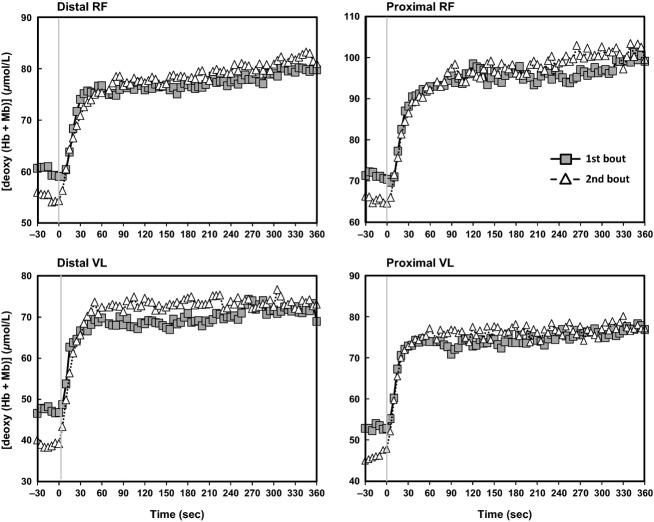
Adipose tissue thickness corrected absolute mean concentrations of deoxyhemoglobin/myoglobin [deoxy (Hb + Mb)] response at each site for first (square) and second (triangle) bouts (error bars are omitted for clarity). A significantly lower baseline at the proximal and distal sites of the vastus lateralis (VL) muscle was present after priming.

Priming heavy exercise significantly increased averaged *τ*_p_ [deoxy (Hb + Mb)] (i.e., slower kinetics) across the four muscle sites (*P* < 0.01) and significantly shortened the TD (Fig.4 and Table2). Regarding the spatial heterogeneity of [deoxy (Hb + Mb)], the intersite TD CVs became significantly greater after priming (*P* < 0.05), while intersite CVs for *τ*_p_ were reduced (*P* < 0.05) and intersite CVs for MRT_p_ unchanged.

**Table 2:** Kinetics of [deoxy (Hb + Mb)] across the four sites and intersite CVs for the primary component at the onset of two bouts of heavy exercise.

	1st bout	2nd bout
TD, *s*	12.5 ± 4.3	9.5 ± 4.6*
*τ*_p_, *s*	11.7 ± 3.8	19.4 ± 7.6**
MRT_p_, *s*	24.1 ± 6.9	28.4 ± 9.0
CV of TD, %	38.7 ± 25.0	62.6 ± 31.9*
CV of *τ*_p_, %	72.7 ± 34.4	49.0 ± 20.9*
CV of MRT_p_, %	39.9 ± 14.3	49.7 ± 29.1

Values are mean ± SD. [deoxy (Hb + Mb)], change in absolute concentration of deoxyhemoglobin + myoglobin

CV, intersite coefficient of variation.

*, ** *P* < 0.05, 0.01, compared to 1st bout.

Baseline [deoxy (Hb + Mb)] was decreased significantly by priming and the amplitude of the response increased across the four muscle sites (*P* < 0.05, Fig.4 and Table3) such that the *A*_a_ (the sum of *A*_p_ + BL) was unchanged. The intersite CV for baseline and *A*_p_ were not significantly different after priming. The proximal RF muscle was characterized by a greater [deoxy (Hb + Mb)] baseline compared with the proximal and distal VL for both exercise bouts. Priming increased (i.e., slowed) *τ*_p_, decreased (i.e., made faster) TD, and decreased BL in both proximal and distal sites of the VL (Table4).

**Table 3:** Amplitude of [deoxy (Hb + Mb)] across the four sites and intersite CVs for the primary component at the onset of two bouts of heavy exercise.

	1st bout	2nd bout
BL, *μ*mol L^−1^	55.0 ± 7.3	50.2 ± 6.7*
*A*_p_, *μ*mol L^−1^	24.5 ± 10.2	30.2 ± 15.9*
*A*_a_, *μ*mol L^−1^	79.5 ± 11.1	78.2 ± 19.2
*A*_i_, *μ*mol L^−1^	−1.4 ± 3.9	0.1 ± 6.7
[deoxy (Hb + Mb)](6-3), *μ*mol L^−1^	2.6 ± 2.6	2.1 ± 4.4
CV of BL, %	19.1 ± 10.4	21.7 ± 10.5
CV of *A*_p_, %	35.7 ± 13.5	30.3 ± 13.7

Vaules are mean ± SD.

*A*_p_, amplitude of primary component

*A*_a_, sum of *A*_p_

BL; *A*_i_, amplitude of initial component; [deoxy (Hb + Mb)](6-3), the slow component calculating as the changes from 3 min to 6 min.

* *P* < 0.05, compared to 1st bout.

**Table 4:** Amplitude and kinetics of [deoxy (Hb + Mb)] and their spatial heterogeneity following the onset of heavy exercise.

	1st bout						2nd bout					
	Baseline, *μ*mol L^−1^	*A*_i_, *μ*mol L^−1^	*A*_p_, *μ*mol L^−1^	TD, *s*	*τ*_p_, *s*	MRT_p_, *s*	Baseline, *μ*mol L^−1^	*A*_i_, *μ*mol L^−1^	*A*_p_, *μ*mol L^−1^	TD, *s*	*τ*_p_, *s*	MRT_p_, *s*
Distal RF	56.8 ± 9.1	−2.6 ± 2.2	20.6 ± 8.0	14.8 ± 6.7	17.9 ± 13.6	32.3 ± 12.8	54.0 ± 7.7	−0.9 ± 4.8	30.1 ± 19.7	8.0 ± 4.3&	22.3 ± 19.2	29.7 ± 20.8
Distal VL	46.1 ± 6.2**	−1.0 ± 2.0	22.8 ± 11.2	13.1 ± 9.1	6.3 ± 2.5	19.3 ± 10.6	40.2 ± 7.0**,#,&	−0.6 ± 2.0	32.7 ± 15.1	9.0 ± 8.1&	19.1 ± 9.6&&	28.1 ± 13.8
Proximal RF	67.9 ± 17.7	−2.2 ± 3.3	28.7 ± 13.0	13.5 ± 6.8	15.1 ± 8.9	29.2 ± 13.0	62.6 ± 15.1	−0.8 ± 3.4	33.3 ± 19.9	15.6 ± 10.6	18.8 ± 11.2	33.6 ± 17.9
Proximal VL	51.9 ± 6.0**	−2.4 ± 4.7	24.8 ± 16.1	9.6 ± 3.9	8.4 ± 6.1	17.9 ± 4.3	45.3 ± 6.8**,&	−0.7 ± 4.4	29.1 ± 15.5	6.7 ± 3.5*	15.1 ± 6.6&	21.8 ± 8.9

Values are mean ± SD (*n* = 12).

*, ** *P* < 0.05, 0.01 versus Proximal RF

# *P* < 0.05 versus Distal RF

&,&& *P* < 0.05, 0.01 versus 1st bout.

### Relationship between [deoxy (Hb + Mb)] heterogeneity and V̇_O2_ kinetics

The priming exercise-induced decrease in the V̇_O2SC_ was not correlated with the altered muscle deoxygenation kinetics (as reflected in the shorter TD and slower [deoxy (Hb + Mb)] response). In addition, the averaged [deoxy (Hb + Mb)] slow component was calculated as the change in [deoxy (Hb + Mb)] from 3 to 6 min across four sites. No correlation was found between the priming-induced small reduction in [deoxy (Hb + Mb)](6-3) and that of V̇_O2_(6-3) (*r* = −0.127, *P* = 0.694).

## Discussion

The original findings of this investigation were consistent with our two hypotheses. First, absolute [total (Hb + Mb)] was increased at baseline (Burnley et al. 2002; DeLorey et al. 2007; Jones et al. 2008) within and among muscles/regions and this response significantly correlated with the reduced V̇_O2SC_. Second, neither the reduction in the baseline [deoxy (Hb + Mb)] nor the reduced intersite CVs of the τ_p_ kinetics correlated with the decreased V̇_O2SC_ or any other differences in the V̇_O2_ response (i.e., reduced MRTp primary component). These results suggest that neither priming-induced increases in the local 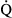
_O2_ relative to V̇_O2_, nor its spatial variation (i.e., intersite CVs of [deoxy (Hb + Mb)] kinetics), contributed to the altered pulmonary V̇_O2_ kinetics. However, the sustained increase in [total (Hb + Mb)] at baseline may, by augmenting D_m_O_2_ and the intracellular consequences of such (i.e., elevated P_intramyocyte_O_2_-induced enhancement of mitochondrial oxidative regulation), reduce the V̇_O2SC._

### Effects of priming on pulmonary V̇_O2_ kinetics

Our finding of the speeded ‘overall’ V̇_O2_ kinetics (reduced TD_p_ and MRT_p_ without change in the primary component *τ*_p_ and reduction in the V̇_O2SC_ amplitude) after prior heavy exercise are consistent with the majority of previous studies (e.g. Bearden and Moffatt 2001; Burnley et al. 2000; Koppo and Bouckaert 2000; MacDonald et al. 1997; Perrey et al. 2003; Scheuermann et al. 2001) with a few exceptions (Bangsbo et al. 2000; Rossiter et al. 2001; Tordi et al. 2003; Paterson et al. 2005; Sahlin et al. 2005). A reduction in the V̇_O2SC_ by itself (i.e., when primary component *τ*_p_ and *A*_p_ are unchanged as in this study) does not reflect a speeding of phase 2 V̇_O2_ kinetics. As previously determined (Gerbino et al. 1996; Jones et al. 2003, 2006) the faster overall V̇_O2_ kinetics occurred consequent to a reduced relative contribution of the V̇_O2SC_ to the overall response.

### Mechanistic basis for reduced V̇_O2SC_

The primary determinant of D_m_O_2_ is considered to be the number of RBCs in the capillary bed adjacent to the contracting muscle fibers at any given instant (Federspiel and Popel 1986; Groebe and Thews 1990). Thus, from Fick's law [V̇_O2SC_ = D_m_O_2_ (P_capillary_O_2_ – P_intramyocyte_O_2_)], for any given V̇_O2,_ the greater the D_m_O_2_ the less capillary-to-myocyte O_2_ pressure differential is required. Capillary (or microvascular) PO_2_ (P_capillary_O_2_) is set by the 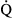
_O2_/V̇_O2_ relationship and, accordingly, if D_m_O_2_ increases, the requisite V̇_O2_ can be achieved with less reduction in P_intramyocyte_O_2_ and hence improved mitochondrial oxidative phosphorylation. The present findings of elevated total [Hb + Mb] after priming, resulting presumably from increased [Hb], provide support for priming exercise elevating D_m_O_2_ which is expected to raise P_intramyocyte_O_2_, thereby “tightening” mitochondrial control (decreasing Δ[PCr], Δ[Pi], Δ[NADH], and Δ[ADP]_free_, (Hogan et al. 1992)) and potentially reducing the V̇_O2SC_.

### Effect of prior heavy exercise on muscle deoxygenation kinetics

The absolute concentrations at BL for [deoxy (Hb + Mb)] at the four different sites before subsequent heavy exercise were significantly reduced compared to the first exercise bout. This extends the findings of Spencer et al. (2012) from single site VL measurements during moderate exercise and supports that local muscle 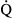
_O2_ was augmented in the presence of elevated muscle V̇_O2_ (DeLorey et al. 2007). Muscle V̇_O2_ is increased for a period following exercise [i.e., excess postexercise O_2_ consumption (EPOC)], while fractional O_2_ extraction is reduced, reflecting a slower decrease in blood flow relative to V̇_O2_ (Ferreira et al. 2006). The present results suggest that the sustained elevations of blood flow (and 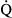
_O2_) during postexercise (recovery) that increase P_microvascular_O_2_ (and P_intramyocyte_O_2_), and additionally may benefit heat and metabolite removal, may be relatively homogeneous. Also, differences in the Bohr effect (i.e., a rightward shift in the O_2_-Hb dissociation curve) due to lactic acidosis (Bhambhani et al. 1997; Grassi et al. 2003) and increases in muscle temperature (Koga et al. 2013) across the different sites could contribute to the reduced spatial and temporal heterogeneity at baseline in the primed muscles. During exercise the macroscopic heterogeneity of O_2_ delivery and V̇_O2_ is undoubtedly impacted by differences in motor unit/muscle recruitment patterns as well as inherent heterogeneities in vascular and metabolic control. To elucidate these effects more fully further studies must exploit technology that can address both microscopic- and macroscopic heterogeneity, that is, the broad extremities of mismatching of O_2_ delivery and V̇_O2_ within and among contracting muscle(s).

### Relationship between heterogeneity of muscle deoxygenation and pulmonary V̇_O2_ kinetics

The diminished [deoxy (Hb + Mb)] baseline prior to the second bout might be attributed in part to the increased bulk and local O_2_ delivery relative to V̇_O2_ (Hernández et al. 2010). However, the *τ*_p_ of the V̇_O2_ kinetics was not decreased from the first to the second bout. This suggests that within and among the muscles, muscle V̇_O2_ kinetics during the primary phase were not dependent on O_2_ delivery (i.e., the condition was to the right of the O_2_ dependency ‘tipping point’) (Poole and Jones 2005; Koga et al. 2007).

There is substantial heterogeneity in muscle blood flow within an active muscle (Whipp et al. 1995; Piiper 2000; Kalliokoski et al. 2006; Heinonen et al. 2007) and the effects of heavy priming exercise on V̇_O2_ (e.g., reduced V̇_O2SC_) might be related to greater homogeneity of 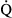
_O2_ distribution and matching to V̇_O2_ (Gerbino et al. 1996; DeLorey et al. 2004, 2007; Jones et al. 2006; Kalliokoski et al. 2006). Indeed, Saitoh et al. (2009) reported that priming reduced the dynamic spatial heterogeneities of muscle deoxygenation matching of 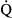
_O2_ to V̇_O2_ within the active muscle (as reflected in the shorter TD and slower [deoxy (Hb + Mb)] kinetics). However in their study, the dynamic spatial heterogeneities of muscle deoxygenation did not correlate with the kinetics of phase II V̇_O2_. Murias et al. (2011) reported that at the reduced moderate intensity, *τ*V̇_O2_ after priming correlated with the reduction in the [deoxy (Hb + Mb)]/V̇_O2_ ratio (proportional to the inverse of 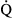
_O2_): The interpretation being that for *τ*V̇_O2_′s >∽20s in a young, healthy population, improved microvascular O_2_ distribution within the active tissues may play an important role in the faster rate of adjustment in V̇_O2_ during moderate exercise following priming. In contrast, the intransigent phase II *τ*V̇_O2_ in the present investigation (∽20s) suggests that the spatial heterogeneity of microvascular 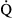
_O2_ (in relation to V̇_O2_) is not related to (i.e., does not determine) the primary phase of V̇_O2_ kinetics during heavy exercise in active young subjects.

### Comparison between RF and VL for the different [deoxy (Hb + Mb)] kinetics

The proximal RF was characterized by a greater baseline [deoxy (Hb + Mb)] for both bouts compared to the VL. Histochemically, RF contains proportionally fewer slow twitch fibers in the surface region compared with the VL (Johnson et al. 1973). Judging from our observations (Table4), the greater baseline [deoxy (Hb + Mb)] in the proximal RF versus the proximal VL is the result of lower 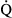
_O2_ relative to V̇_O2_ (i.e., higher V̇_O2_/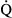
_O2_). Interestingly, Chin et al. (2011) reported that, compared with the VL, the RF [deoxy (Hb + Mb)] kinetics evidenced a “right-shifted” response throughout ramp exercise. This may be evidence for a lower activation (supported by EMG) rather than a higher 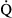
_O2_/V̇_O2_ at a given V̇_O2_ especially as muscles comprised of fast twitch fibers typically have far lower 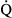
_O2_/V̇_O2_ (and thus lower P_microvascular_O_2_) than their slow twitch counterparts (Richardson et al. 1998; Behnke et al. 2002; McDonough et al. 2005). The V̇_O2SC_ is linked either directly or indirectly to the recruitment of type II muscle fibers at higher work rates and/or to metabolic changes occurring within the initially recruited fibers (Grassi et al. 2015). Furthermore, it is thought that the availability of O_2_ may play an important role in regulating the recruitment of these high-threshold type II motor units (Bearden and Moffatt 2001; Jones et al. 2011; MacDonald et al. 1997; McDonough et al. 2005; Piiper 2000; Saitoh et al. 2009). Thus, it is possible that the shorter TD, the slower *τ*, and/or the decreased spatial heterogeneity of muscle deoxygenation kinetics (as the primary component *τ*) in the second bout reflected an improved distribution of local 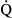 and matching of muscle 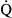
_O2_ to muscle V̇_O2_ in those fibers recruited early into exercise which may have delayed their fatigue, thereby reducing the recruitment of more type II fibers. Therefore, the greater intracellular PO_2_ (as reflected in the greater muscle 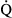
_O2_/V̇_O2_, detected as lower [deoxy (Hb + Mb)]), during the primary component might be related to the reduction in the SC. Alternatively, the changes in muscle deoxygenation kinetics might have decreased the degree of metabolic perturbation, implicated in the V̇_O2SC_, within already recruited fibers.

In conclusion, the relationship between priming-induced increase in baseline [total (Hb + Mb)] (Burnley et al. 2002; DeLorey et al. 2007; Jones et al. 2008) within and among muscles/regions and the reduced V̇_O2SC_ suggests that resultant enhancements of D_m_O_2_ may be responsible for the overall faster V̇_O2_ kinetics. Augmented D_m_O_2_ would be expected to raise P_intramyocyte_O_2_ and thereby enhance mitochondrial oxidative regulation and reduce V̇_O2SC_. The temporal displacement between increased [total (Hb + Mb)] at baseline and reduction in V̇_O2SC_ implies that better oxygenation during the initial fast component (phase II) kinetics relates quantitatively to subsequently improved metabolic control. In contrast, neither the degree of reduction in baseline [deoxy (Hb + Mb)] nor the CVs of TD, and *τ*_p_ of [deoxy (Hb + Mb)] across the four sites (i.e., spatial variation) were related to the overall faster pulmonary V̇_O2_ kinetics. Indeed, our present results in combination with those of Saitoh et al. (2009) demonstrate that reduced heterogeneity of muscle deoxygenation is not requisite for priming exercise to speed overall V̇_O2_ kinetics. Further studies might usefully focus upon the extent to which increased or decreased heterogeneity is beneficial/detrimental under physiological and/or pathophysiological conditions.

## Conflict of Interest

None declared.
